# Combination of Healthy Lifestyle Factors on the Risk of Hypertension in a Large Cohort of French Adults

**DOI:** 10.3390/nu11071687

**Published:** 2019-07-23

**Authors:** Helene Lelong, Jacques Blacher, Julia Baudry, Solia Adriouch, Pilar Galan, Leopold Fezeu, Serge Hercberg, Emmanuelle Kesse-Guyot

**Affiliations:** 1 AP-HP, Diagnosis and Therapeutic Center, Faculty of Medicine, Hôtel-Dieu Hospital, Paris-Descartes University, 75004 Paris, France; 2 UREN (Nutritional Epidemiology Research Unit)—U557 INSERM, U1125 INRA, CNAM, CRNH IdF, Paris 13, Sorbonne Paris Cité University, 93000 Bobigny, France; 3 Department of Public Health, Avicenne Hospital, 93000 Bobigny, France

**Keywords:** hypertension, epidemiology, lifestyle, nutrition

## Abstract

Background: Healthy lifestyle factors are widely recommended for hypertension prevention and control. Nevertheless, little is known about their combined impact on hypertension, in the general population. Our aim was to compute a Healthy Lifestyle Index (HLI) comprising the main non-pharmacological measures usually recommended to improve hypertension prevention: normal weight, regular physical activity, limited alcohol consumption, adoption of a healthy diet; to evaluate their combined impact on hypertension incidence. Methods: We prospectively followed the incidence of hypertension among 80,426 French adults participating in the NutriNet-Santé cohort study. Self-reported dietary, socio-demographic, lifestyle and health data were assessed at baseline and yearly using a dedicated website; the association between HLI and hypertension risk was assessed by multivariable Cox proportional hazards models adjusted for age, sex, family history of hypertension, socio-demographic and lifestyle factors. Hypothetical Population Attributable Risks associated to each factor were estimated. Results: During a median follow-up of 3.5 years (IQR: 1.5–5.3), 2413 incident cases of hypertension were identified. Compared with no or one healthy lifestyle factor, the hazard ratios (HR) for hypertension were 0.76 (95% CI, 0.67–0.85) for two factors, 0.47 (95% CI, 0.42–0.53) for three factors and 0.35 (95% CI, 0.30–0.41) for all healthy lifestyle factors (*p*-trend <0.0001). Compared with adhering to 0, 1, 2 or 3 healthy lifestyles, adhering to all of them was found associated with a reduction of the hypertension risk of half (HR = 0.55 (95% CI, 0.46–0.65)). Conclusion: Active promotion of healthy lifestyle factors at population level is a key leverage to fight the hypertension epidemic.

## 1. Introduction

Recent data reported hypertension as the most important contributor to the global burden of disease and to global mortality, leading to 9.4 million deaths each year [1]. Moreover, considering the increasing prevalence of the condition, primary prevention would have a major beneficial impact in terms of public health. French as well as international guidelines claim appropriate lifestyle changes as the cornerstone for the prevention of hypertension as they may safely prevent or delay hypertension in non-hypertensive subjects [2,3,4]. Overweight, unhealthier diet (including excessive consumption of salt), alcohol consumption and low levels of physical activity have been identified as the most important modifiable risk factors for hypertension. Whereas concerning dietary, we reported in a previous study, that Adherence to the Dietary Approach to Stop Hypertension (DASH) could reduce or delay hypertension incidence [5].

Despite the individual effects of specific lifestyle changes that have been reported through randomized trials, little is known about their combined effect on hypertension incidence on a general population. Two studies focused on it, the first, conducted in a population of female nurses in the United States, and the second in a population of Chinese individuals, both reported that adopting healthy lifestyle factors could prevent a large proportion of new onset hypertension [6,7]. Furthermore, in recent years, combined impact of healthy lifestyle factors has been investigated in cancer research area, in particular in the European Prospective Investigation into Cancer and Nutrition EPIC Cohort, through the development of a healthy lifestyle index (HLI) composed of potentially modifiable lifestyle factors associated with colorectal cancer [8].

The aim of the present study was firstly to compute a HLI comprising the identified modifiable factors related to hypertension and to investigate their combined effect on the incidence of hypertension and secondly to estimate the population attributable risks [9], of individuals and combined modifiable risk factors, as well as addressing the percentage of new hypertension cases that would be hypothetically avoided in a large cohort of healthy French adults.

## 2. Methods

### 2.1. Study Design and Population

The NutriNet-Santé cohort Study is an ongoing French web-based study, launched in May 2009 to investigate the relationship between nutrition and chronic diseases. The study’s protocol, design and methods have been described in detail in a previous publication [10]. In summary, the NutriNet-Santé Study was implemented in a general population, targeting Internet-using adult volunteers recruited by a large multimedia campaign. Using a dedicated website, volunteers were asked to fill out questionnaires at baseline and yearly, via an online website reporting information on socio-demographics, lifestyle characteristics, health status, and diet. All participants signed an informed consent form. The NutriNet-Santé Study was approved by the International Research Board of the French Institute for Health and Medical Research (0000388FWA00005831) and the” Comité National de l’Informatique et des Libertés” (CNIL n° 908450 and n° 909216).

Sampling of the present analysis was fully described in a previous study [5]. Between May 2009 and October 2015, 140, 001 participants were included in the cohort. Only those with 3 validated 24-Hour dietary Records (24 HR) where selected: therefore, 16,570 participants were not selected because they were identified as energy under-reporters based on the method developed by Black [11] and 26,998 because they did not complete three 24 HR. We secondly excluded participants with hypertension at baseline (prevalent cases), cancer, diabetes, cardiovascular disease and pregnant women (*n* = 11,731), lastly, we excluded participants with missing health data, anthropometrics measurements or physical activity (*n* = 4,276) data. Consequently, 80,426 participants were included in the present analyses (see Figure S1 in supplemental materials).

### 2.2. Case Ascertainment

Health questionnaire provided personal and family history of hypertension (defined as having at least one hypertensive first-degree parent) and medication use. During the follow-up, annual questionnaires enabled the identification of 2413 incident cases of hypertension. New hypertension cases were based on self-reported diagnosis and/or use of anti-hypertensive therapy. In the cohort, 73% of case ascertainments were based on self-reported diagnosis of hypertension and antihypertensive therapy, 7% were based on self-reported diagnosis of hypertension only, and 20% were based on adequate antihypertensive therapy reported without having declared other pathology requiring such medication.

### 2.3. Assessment of Lifestyle Factors

#### 2.3.1. Dietary Intake Assessment

At inclusion, participants completed three 24-h dietary records (24 HR) randomly allocated over a two-week period, including two weekdays and one weekend day; this collection method has shown high agreement with the reference method (interview with a dietician) [12] and was validated against biomarkers [13,14]. Participants reported all foods and beverages consumed at each meal. The NutriNet-Santé food composition table, which includes more than 3000 food items, enabled to estimate nutrient intakes [15]. Portion sizes were either estimated with photographs, derived from a validated picture booklet [16], or quantity consumed was directly entered. Daily dietary intakes were calculated as the weighted average from the three 24 HR. A frequency questionnaire on the amount and type of alcohol consumed was used to calculate alcohol intake (g ethanol/day). Based on these nutritional data, we have computed a DASH-Style Diet score as previously developed by Fung et al. [17]; including 8 dietary components whose consumption should be increased (fruits, vegetables, nuts and legumes, low-fat dairy, whole grains) or minimized (sodium, sweetened beverages, red and processed meats). Gender–specific (ranging from 1 to 5) quintiles were used for individual’s sub scores in each dietary component. Thus, final DASH score ranging (8 to 40 points) was calculated by adding all the sub scores.

#### 2.3.2. Demographic, Anthropometrics, and Lifestyle Data Collection

Baseline questionnaires provided data on socio-economic status: educational level was reported in three categories referred to the highest achieved diploma (primary school, high school and University or equivalent); smoking status was reported in three categories (current smoker, never smoker and former smoker); self-reported weight and height (in order to calculate the Body Mass Index (BMI) by dividing weight, in kilograms, by height, in square meters). Physical activity (PA) was assessed using the short form of the International Physical Activity Questionnaire (sf-IPAQ), in the French language, collecting information on frequency, duration and intensity of PA [18]. This questionnaire allows estimating weekly energy expenditure, expressed in metabolic equivalent (MET) *h/week as a continuous variable (met/h/week).

### 2.4. HLI Definition

Regarding international guidelines, four modifiable lifestyle factors known to be associated with hypertension risk were selected [3,4]: weight reduction or maintenance, regular physical exercise, moderation of alcohol consumption, adoption of a DASH type diet including limited salt consumption. For each of these components, a binary variable was created with 1 point allocated to low risk group (0 otherwise) defined as follows: having a healthy weight i.e., a BMI of less than 25, which is the current cut point defined by the World Health Organization [19,20]; having regular physical activity, i.e., at least 30 min of brisk walking/day [2,3,21]; having a limited alcohol consumption to no more than 20 g of ethanol per day as [2,3,4,22]; having a healthy diet, i.e., a DASH score in the top quartile which include a limited salt consumption as described in the section above [2,3,4,5,23,24]. The HLI was then calculated as the sum of the binary variables leading to an HLI which ranged from 0 to 4.

### 2.5. Statistical Analysis

We performed descriptive analyses on the prevalence of each component included in the HLI in our sample and characteristics of participants by HLI. We used Cox proportional hazards models to provide hazards ratios (HRs) and 95% confidence intervals (CIs) to estimate the association between hypertension incidence and the modifiable risk factors individually and in combination through the HLI. Age was used as primary time-dependent variable. Participants contributed person-time until age at diagnosis of HTA, death or last completed health follow-up questionnaire, whichever occurred first. The assumptions of proportionality were checked through examination of the log–log (survival) versus log-time plots. Associations of the modifiable risk factors with hypertension incidence were estimated for each modifiable risk factor modeled as a binary variable with 0 points as reference group. The first model was adjusted for age (time-scale), sex, educational level, smoking status and familial history of hypertension. The second model was additionally adjusted for the other HLI components.

To estimate the association of the modifiable risk factors in combination, HLI was modeled as an ordinal variable according to four categories: 0 or 1 (reference group); 2; 3 and 4. We also estimated the HR of participants meeting the low risk category for the 4 HLI components compared to others. Models were adjusted for age (time-scale), sex, educational level, smoking status and familial history of hypertension.

Interactions between sex and each component and HLI on hypertension were tested. No significant interaction was detected thus analyses were performed in the overall sample.

However, we performed subgroup analyses in the three BMI categories (normal weight: BMI < 25 kg/m^2^; overweight: 25 ≤ BMI < 30 kg/m^2^ and obese: BMI ≥ 30 kg/m^2^) since body weight may be considered as a consequence of the other modifiable risk factors. Furthermore, as hypertension has a multi-factorial origin, including genetic and lifestyle factors, we performed sensibility analyses stratifying by family history of hypertension to investigate whether the association with HLI would be similar among those with and without a genetic predisposition [25].

Concerning alcohol, guidelines’ recommendation is to limit the consumption among men and women as the relationship between alcohol consumption and blood pressure would be linear [3,4]. However, some data reported that, in women, this relationship would be J-shapped [26,27] and low-risk alcohol intake would therefore be defined as greater than zero but not exceeding 10 g/day. We have therefore performed sensitivity analyses to investigate the association of alcohol intake in hypertension risk, considering as low risk group an alcohol consumption not exceeding 20 g/day in men and greater than zero but not exceeding 10 g/day in women.

Finally, we calculated the hypothetical percentage of population attributable risks (PARs) and 95% CIs to estimate the proportion of hypertension cases attributed to not meeting the recommendations. PARs were calculated using the standard equation from Miettinen [28]:

PAR= pE|D (RR − 1)/RR(1)

where pE|D is the prevalence of individuals in the exposed group (here individuals not in the low risk group) and RR the associated multivariable-adjusted ratio obtained by reversing the coding of the protective factor. Upper and lower CIs of the PARs were estimated by bootstrapping with 500 replications.

All tests were two-sided, *p* < 0.05 was considered statistically significant. Statistical analyses were performed using SAS software version 9.2 (SAS Institute, Cary, NC, USA).

## 3. Results

During a median follow-up of 3.5 years (IQR, 1.5–5.3), 2413 subjects out of 80,426, reported a new diagnosis of hypertension. Mean age at inclusion was 41.9 ± 14.0 years and 20% were men.

Prevalence of the modifiable risk factors in the sample is available in Table 1. Table 2 summarizes participants’ characteristics at baseline according HLI (in five categories). Participants with greater HLI were mainly women, had a higher level of education and lower tobacco consumption.

For each modifiable risk factor, meeting the low risk group was associated with a significant reduction in risk of hypertension after adjustment for age, sex, educational level, tobacco consumption and familial history of hypertension. When all the components were introduced in the model, having a healthy weight and a healthy diet remained statistically significant with reduction of risk of hypertension of 54 % and 28% respectively. Nevertheless, there was an indication of an association but no significant independent risk reduction for low risk group of physical activity or alcohol consumption after additional accounting for all modifiable risk factors (Table 3).

**Figure 1:**
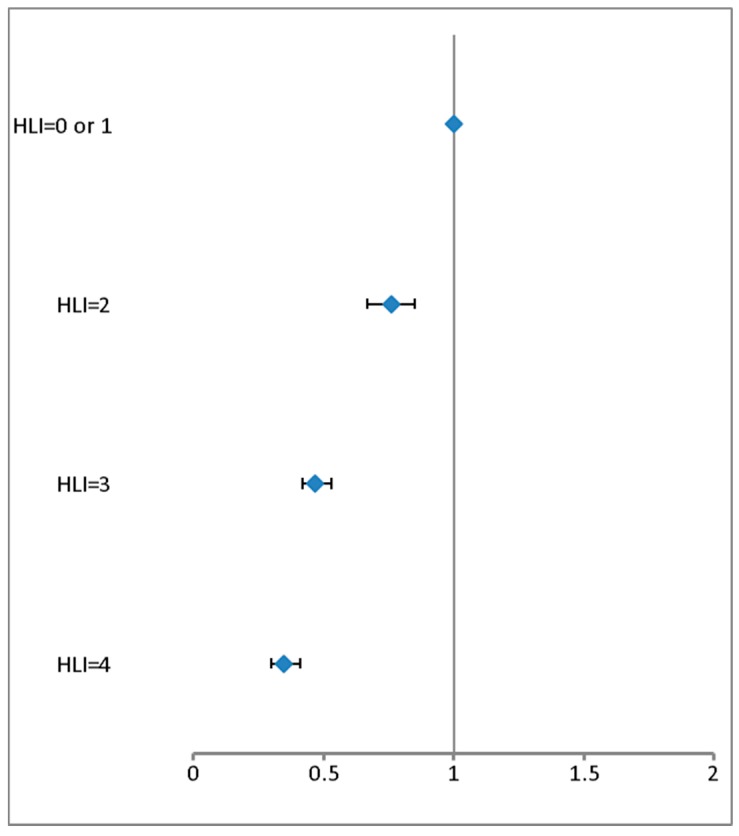
Multivariable-adjusted hazard ratios of incident Hypertension according to increasing number of Healthy Lifestyle Factors (HLI).

Figure 1 reports the relationship between combination of modifiable risk factors through the HLI and risk of incidence of hypertension. Compared with having 0 or 1 healthy factor, each additional low-risk factor was associated with a greater risk reduction; and we reported a 31% lower risk of hypertension for one-point increase on the HLI. Participants who met all the healthier categories for all the modifiable risk factors have a 44% decreased risk of hypertension, compared with those who did not (see Table S1 in supplemental materials).

Hypothesized percent of PARs corresponding to each individual modifiable risk factor and their combination are reported in Table 4. Under the causality assumption, more than one quarter of the new cases of hypertension were attributable to being overweight; more than 20% to unhealthier diet, 1% to low physical activity and 0.5% to excessive alcohol consumption. Almost 40% of the new cases of hypertension would be attributable to not adhering to all recommendations.

Sensitivity analyses considering an alcohol consumption not excessing 20 g/day in men and greater than zero but not excessing 10 g/day in women as low risk group showed similar results.

Sensitivity analyses stratifying by BMI categories reported similar reduction in risk of hypertension in link with combination of healthy diet, physical activity and alcohol consumption, in normal weight (*n* = 59,468; cases number = 1232, HR = 0.76 (95%CI, 0.67–0.87)) and in overweight (*n* = 15,466; cases number = 780 HR = 0.75 (95%CI, 0.61–0.92)), but in the obese category (*n* = 5492 and cases number = 401), meeting the three low risk groups was associated with a less strong reduced risk of hypertension and the association was not significant: HR = 0.90 (95%CI, 0.66–1.12).

Stratified analyses by family history of hypertension reported similar results in participants with or without familial predisposition either for each individual factor or for the HLI (data not shown).

## 4. Discussion

In this large cohort of French adults issued from the general population, healthy lifestyle factors advocated in guidelines to prevent and manage hypertension, individually or in combination, should avoid a large number of new cases of hypertension, the most prevalent chronic disease worldwide.

### 4.1. Effect of Individual Factors

We found healthy weight as the strongest protective factor of hypertension. The positive association between weight and blood pressure as well as hypertension, has been fully documented not only in epidemiologic studies, conducted in several populations [6,7,29,30,31] (including French population samples [32,33]), but also in experimental studies with a meta-analysis reporting a 4 mmHg mean reduction in systolic and diastolic blood pressure with an average weight loss of 5.1 kg [34]. Our results are in agreement with this body of evidence, with 26% of observed new cases of hypertension hypothetically due to overweight and obesity reasserting the link between epidemics of hypertension and obesity. Stratified analyses by BMI categories reported that combination of low risk factors was similarly associated with a reduced risk of hypertension in normal and overweight categories but not in obese. These results suggest that obese people could be resistant to the positive effect of appropriate lifestyles and switch to adherence should be efficient before this stage. However, because of the small number of obese participants, in particular obese participants with three low risk factors, low power may partly explain the non-significant association.

In our population, we found that more than 20% of new onset of hypertension would be hypothetically due to an unhealthy diet. This finding was in accordance with scientific literature emphasizing the protective effect of DASH-diet to prevent hypertension and rise of BP trough prospective studies [24,35], and many interventions trials [36]. Our results underline the potential benefit to improving diet in the global population.

In our study, effect of low risk alcohol consumption or physical activity on risk of hypertension was less prominent compared to those of BMI and diet. Concerning alcohol consumption, we elected to consider as high risk excessive consumption but not abstinence, considering that in the public health perspective, it is not relevant to promote alcohol consumption among nondrinkers. In this way, we did not take into account the potential protective effect of moderate consumption previously described in women [26,27]. However, sensitivity analyses by modifying the definition of the high-risk category provided similar results and thus do not support a potential protective effect. It is important to note that, in our sample, alcohol consumption was moderate which could explain the modest effect we observed with limited statistical power to detect and effect of high consumption. Anyway, even if only a small proportion of new hypertension cases could be avoided by limiting alcohol consumption and practicing regular physical activity, this could still have a major benefit in terms of public health (at the population level), given the high prevalence of hypertension.

Combination of modifiable risk factors. Besides individual effect of modifiable risk factors, we found that adherence to a combination of them could avoid a very large proportion of new cases of hypertension. In accordance with the results reported by Forman et al. in women [6], this combined effect was beneficial both in participants with and without family history of hypertension, suggesting that appropriate lifestyle could at least delay the onset of the condition in predisposed persons.

### 4.2. Limitations and Strengths

The present study has some limitations. First, biases related to the data collection: a selection bias, as excluded subjects for missing data could be different from the included, but also a misclassification bias was possible since the outcome as well as exposure data were both self-reported; however, concerning the outcome, antihypertensive treatment was concomitantly reported by most volunteers who reported being newly hypertensive during follow-up.

Moreover, in the Nutrinet-Santé study, several validation studies on sub-samples have reported high concordance between self-reported anthropometrics data and measured data taken during medical visit by trained technicians using standardized protocol (concordance for BMI classification was 93%) [37]; and dietary data were assessed with repeated 24 HR that are known to provide more accurate estimates of individuals’ intakes than food frequency questionnaire [38] and validated against urinary and blood markers [13,14]. Second, the dichotomization of each factor with a relatively arbitrary threshold (although chosen according to the guidelines), could have led to a loss of information (limiting statistical power) and or a misjudgment of the true effect, but several sensitivity analyses have been conducted to explore the robustness of our findings. Third, the external validity of our result could be limited as the selected sample was not representative of the French general population. Previous publications on the subject reported that women and well-educated individuals were over-represented in the cohort [39] and moreover that volunteers exhibited healthier dietary habits, notably intakes of alcohol beverages were lower in the cohort compared with the general population [40]. Fourth, although we refer to the international guideline, we probably did not include other potential confounding factors that could additionally influence the risk of hypertension. 

However, the NutriNet-Santé study presents several strengths such as the important size of the cohort issued from the general population, the prospective design, the quality and the wide range of the data. Moreover, estimation of health impact measures, such as PARs, could help in quantifying health decisions impacts.

## 5. Conclusions

In conclusion, in our large cohort of French adults, combined lifestyle factors, i.e., regular physical activity and limited alcohol consumption in addition to the well-known protective factors of healthy weight and healthy diet, are associated with a lower hypertension incidence. Moreover, if implementation of all appropriate lifestyle changes appears to be difficult in real life, our findings suggest that each additional adoption of a healthy factor could be protective against new onset of hypertension. Finally, active promotion of healthy lifestyle would have major public health benefits.

## Figures and Tables

**Table 1:** Description and prevalence of the components of the Healthy Lifestyle Index (HLI).

Modifiable Risk Factor	Index Point	Description	Prevalence (%)
**Weight**	0	Overweight (BMI ≥ 25)	26.1
	1	Normal weight (BMI < 25)	73.9
**Physical Activity**	0	Less than 30 min/day of brisk walking	24.2
	1	Equivalent or more than 30 min/day of brisk walking	75.8
**DASH Diet**	0	DASH score lower than the fourth quartile value	76.0
	1	DASH score in the fourth quartile	24.0
**Alcohol Consumption**	0	Excessive Alcohol Consumption (>20 g/day)	9.5
	1	Limited Alcohol Consumption (≤20 g/day)	90.5

BMI: Body Mass Index; DASH, Dietary Approach to Stop Hypertension.

**Table 2:** Baseline characteristics of participants by Healthy Lifestyle Index (HLI) score.

Characteristics	HLI				
	0	1	2	3	4
Participants *n* (%)	541 (0.7)	7219 (8.9)	24,800 (30.8)	35,749 (44.5)	12,117 (15.1)
No. of hypertension cases (%)	37 (6.8)	391 (5.4)	889 (3.6)	810 (2.3)	286 (2.4)
Age (years)	48.3 ± 12.9	44.0 ± 13.3	41.3 ± 13.7	40.6 ± 14.1	45.5 ± 14.1
%men	60.6	32.4	21.6	17.2	16.5
Family history of hypertension	28.1	32.8	30.9	29.9	32.6
Education Level (%)					
Primary	5.0	3.4	2.5	2.0	1.7
High school	31.2	36.4	33.1	30.5	26.2
University or equivalent	63.8	60.2	64.4	67.5	72.1
Smoking status (%)					
Never smoker	29.9	40.3	48.0	54.5	56.7
Former smoker	42.5	37.9	32.6	29.2	33.5
Current smoker	27.5	21.8	19.4	16.4	9.8
BMI (kg/m^2^)	28.3 ± 3.4	28.1 ± 4.5	24.9 ± 4.7	22 ± 2.8	21.2 ± 2
Physical Activity (meth/h/week)	745 ±783	1344 ±2,046	2169 ±2,494	3054 ±2,624	3481 ±2,715
Alcohol intake (g/day)	36.4 ± 18	15.6 ± 18.1	8.9 ± 12.2	5.5 ± 6.6	4.4 ± 4.9
DASH score (mean±SD)	21.2 ± 3.8	21.3 ± 3.8	22.1 ± 4	23.5 ± 4.4	30.4 ± 2.2
DASH score components					
Fruits and vegetables (g/day)	391.7 ± 219.8	392.9 ± 208.5	411.7 ± 212	453.6 ± 225.1	669.7 ± 267.5
Whole-grain (g/day)	19.2 ± 31.4	22.1 ± 37	24.8 ± 39.9	31.8 ± 47	71.1 ± 63.5
Legumes (g/day)	10.5 ± 24	9.4 ± 22.9	9.9 ± 23.7	11.2 ± 25.7	22.9 ± 38.6
Low-fat dairy (g/day)	176.4 ± 145.7	183.3 ± 148.7	186.5 ± 148.4	191.9 ± 151.3	201.2 ± 160.3
Nuts (g/day)	4.3 ± 11.9	3 ± 9.1	3.2 ± 9.2	4.2 ± 10.7	12.2 ± 20.3
Meat (g/day)	103 ± 62.9	89.2 ± 61.2	74.7 ± 54.4	62.4 ± 48.9	30.3 ± 34.3
Soda (mL/day)	49.6 ± 117.1	58.0 ± 125.5	58.0 ± 122.6	53.3 ± 115	10.9 ± 39.8
Sodium (mg/day)	3246 ± 1128	2953 ± 1008	2772 ± 942	2642 ± 880	2419 ± 841

**Table 3:** Hazard ratios of incident hypertension in relation to individual lifestyle factors.

Healthy Lifestyle Factor	Index	No. of Hypertension Cases	HR (95%CI) ^a^	HR (95%CI) ^b^
Overweight	0	1181	1 (ref)	1 (ref)
	1	1232	0.45 (0.41–0.48)	0.46 (0.43−0.50)
Low Physical Activity	0	520	1 (ref)	1 (ref)
	1	1893	0.86 (0.78−0.95)	0.96 (0.8−1.01)
DASH score lower than the top quartile value	0	1839	1 (ref)	1 (ref)
	1	574	0.66 (0.60−0.72)	0.72 (0.66−0.80)
Excessive Alcohol consumption	0	369	1 (ref)	1 (ref)
	1	2044	0.91 (0.88−1.00)	0.98 (0.89−1.04)

HRs, Hazard Ratios; 95% CI, 95%Confidence Intervals; PA, Physical Activity; DASH, Dietary Approach to Stop Hypertension. ^a^ model1: adjusted for age (as primary time dependent variable), sex, educational level, smoking and family history of hypertension. ^b^ model2:model1 additionally adjusted for other modifiable factors: overweight and obesity, physical activity, alcohol consumption, and diet quality.

**Table 4:** Hypothesized percent of Population Attributable Risks (PARs) according to individual modifiable risk factors and combination.

	No. of Hypertension Cases	%PAR (95% CI) ^a^
Overweight	1181	26 (23 to 29)
Low Physical Activity	520	-
DASH score in the third quartile or lower	1839	21 (15 to 27)
Excessive Alcohol consumption	369	-
HLI < 4	2127	39 (32 to 46)

PARs, Population Attributable Risks; PA, Physical Activity; DASH, Dietary Approach to Stop Hypertension; Healthy Lifestyle Index (HLI). ^a^ PARs were calculated using the standard equation from Miettinen [26] from multivariable-adjusted hazard ratios in relation to individual lifestyle factors. Upper and lower CIs of the PARs were estimated by bootstrapping. Cox proportional hazards models were used to provide hazards ratios (HRs) and 95% confidence intervals (CIs) adjusted for age (as primary time dependent variable), sex, educational level, smoking and family history of hypertension. ^a^ linear trends was tested using HLI as ordinal variable.

## Supplementary Materials

The following are available online at https://www.mdpi.com/2072-6643/11/7/1687/s1, Figure S1: Flow chart, Table S1: Hazard ratios of incident hypertension in relation to Healthy Lifestyle Index (HLI).
