# Supplementary material for: Combination of Healthy Lifestyle Factors on the Risk of Hypertension in a Large Cohort of French Adults
Source: Nutrients. 2019 Jul 23;11(7):1687. doi: 10.3390/nu11071687 (PMC6683281; doi:10.3390/nu11071687)
Supplement: Supplementary file 1 [file nutrients-11-01687-s001.pdf]

Figure S1: Flow chart

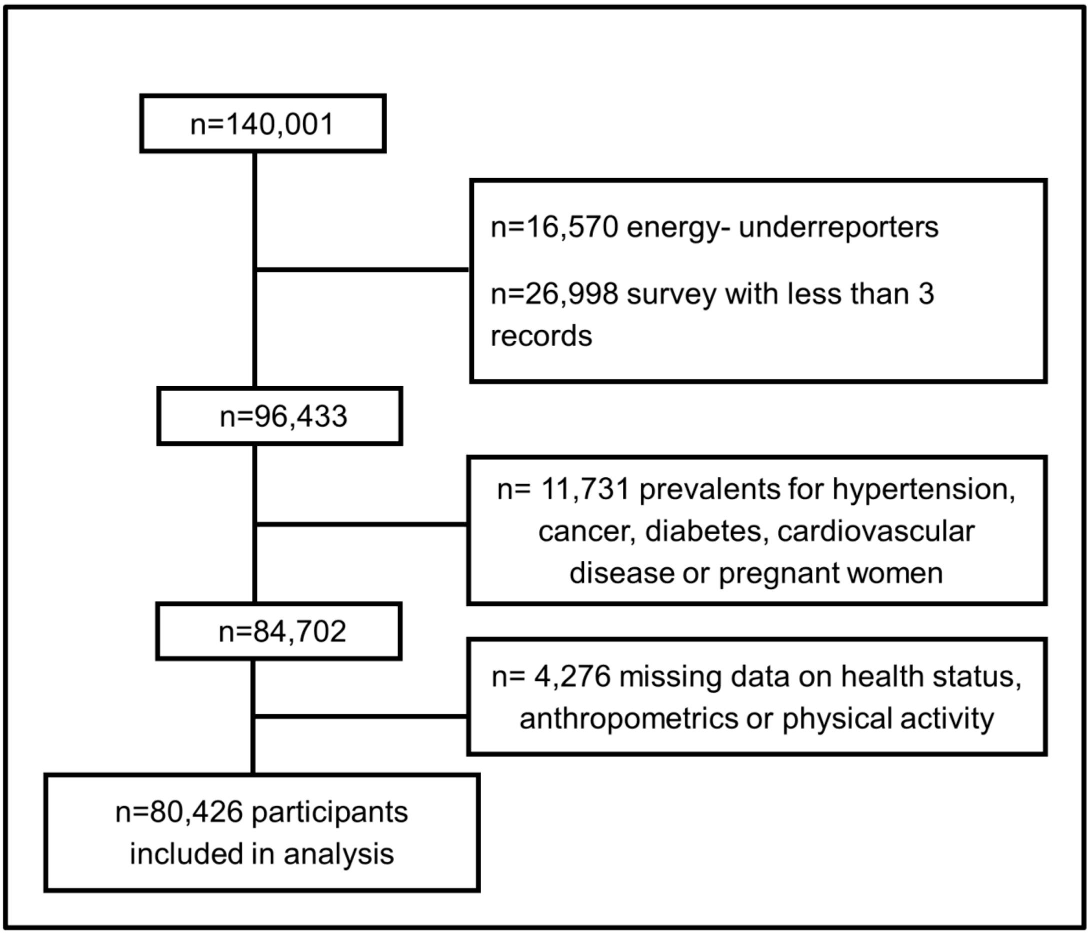

Table S1: Hazard ratios of incident hypertension in relation to Healthy Lifestyle Index (HLI).

HLI	Cases number	HR (95%CI)[a]	p-value
0 or 1	428	1 (ref)	<0.0001[b]
2	889	0.76 (0.67-0.85)	
3	810	0.47 (0.42-0.53)	
4	286	0.35 (0.30-0.41)	
Per one point increase	-	0.69 (0.66-0.72)	<0.0001[b]
HLI<4	2127	1 (ref)	<0.0001[c]
HLI=4	286	0.56 (0.49-0.65)	

[a] adjusted for age (as primary time dependent variable) , sex, educational level, smoking and family history of hypertension

[b] linear trends were tested using HLI as ordinal variable

[c] p-value was calculated using the Wald test.
